# Natural Language Processing and Machine Learning for Identifying Incident Stroke From Electronic Health Records: Algorithm Development and Validation

**DOI:** 10.2196/22951

**Published:** 2021-03-08

**Authors:** Yiqing Zhao, Sunyang Fu, Suzette J Bielinski, Paul A Decker, Alanna M Chamberlain, Veronique L Roger, Hongfang Liu, Nicholas B Larson

**Affiliations:** 1 Department of Health Sciences Research Mayo Clinic Rochester, MN United States

**Keywords:** stroke, natural language processing, electronic health records, machine learning

## Abstract

**Background:**

Stroke is an important clinical outcome in cardiovascular research. However, the ascertainment of incident stroke is typically accomplished via time-consuming manual chart abstraction. Current phenotyping efforts using electronic health records for stroke focus on case ascertainment rather than incident disease, which requires knowledge of the temporal sequence of events.

**Objective:**

The aim of this study was to develop a machine learning–based phenotyping algorithm for incident stroke ascertainment based on diagnosis codes, procedure codes, and clinical concepts extracted from clinical notes using natural language processing.

**Methods:**

The algorithm was trained and validated using an existing epidemiology cohort consisting of 4914 patients with atrial fibrillation (AF) with manually curated incident stroke events. Various combinations of feature sets and machine learning classifiers were compared. Using a heuristic rule based on the composition of concepts and codes, we further detected the stroke subtype (ischemic stroke/transient ischemic attack or hemorrhagic stroke) of each identified stroke. The algorithm was further validated using a cohort (n=150) stratified sampled from a population in Olmsted County, Minnesota (N=74,314).

**Results:**

Among the 4914 patients with AF, 740 had validated incident stroke events. The best-performing stroke phenotyping algorithm used clinical concepts, diagnosis codes, and procedure codes as features in a random forest classifier. Among patients with stroke codes in the general population sample, the best-performing model achieved a positive predictive value of 86% (43/50; 95% CI 0.74-0.93) and a negative predictive value of 96% (96/100). For subtype identification, we achieved an accuracy of 83% in the AF cohort and 80% in the general population sample.

**Conclusions:**

We developed and validated a machine learning–based algorithm that performed well for identifying incident stroke and for determining type of stroke. The algorithm also performed well on a sample from a general population, further demonstrating its generalizability and potential for adoption by other institutions.

## Introduction

Stroke is a syndrome involving a rapid loss of cerebral function with vascular origin [1]. The loss of function can result in deep coma or subarachnoid hemorrhage. There are two broad categories of stroke: hemorrhagic and ischemic stroke [2]. Hemorrhage is caused by bleeding within the skull cavity, while ischemia is characterized by inadequate blood to supply a part of the brain. Stroke identification is an important outcome for various cardiovascular studies [3-5]. However, a challenge with stroke ascertainment is the inconsistent use of International Classification of Diseases (ICD) codes [6], which may result in inaccurate code-based ascertainment of cases [7]. Therefore, the time-consuming process of electronic health record (EHR) abstraction remains the gold standard of stroke ascertainment [8,9].

Machine learning has recently gained popularity for its ability to classify patients or make predictions on various aspects of diseases. In contrast to manually curated algorithms based on domain expertise, machine learning is a data-driven approach that can be trained on large data sets to identify and leverage complex feature relationships and improve classification and prediction tasks thereby. In terms of stroke, machine learning algorithms have been applied to predict future stroke cases [10], mortality and recurrent strokes [11,12], and treatment outcomes [13,14]. Most existing phenotyping algorithms have been developed to only differentiate between cases and noncases of diseases [15-18]; however, ascertaining incident disease (ie, first occurrence of disease) in a population is a more difficult task [8,19,20]. A recent study by Ni et al [21] examined potential predictive features of stroke occurrence including demographic, clinical, and diagnostic characteristics of patients. The authors found that diagnostic tests for stroke, such as computed tomography (CT) and magnetic resonance imaging (MRI), contributed to most of the model performance, and that the optimal feature set included imaging findings, signs and symptoms, interventions, emergency department assessments, findings from angiography and carotid ultrasound tests, ICD codes, substance use (smoking, alcohol, and street drugs) characteristics, and demographics. However, features such as signs and symptoms, substance use characteristics, and demographics may not be specific enough for disease ascertainment, as there is a high prevalence of strokelike symptoms among people without a diagnosis of stroke [22]. In addition, incorporating too many features in the model may result in overfitting without appropriate regularization. Another study [7] also used ICD and Current Procedural Terminology (CPT) [23] codes as features to classify positive, possible, and negative stroke cases. However, stroke-related clinical concepts (including both disease name concepts and symptom concepts) in unstructured clinical notes were not included in this model.

Rapid adoption of EHRs has enabled secondary use of the EHR data in epidemiological research [24-26]. Previous studies noted the existence of bias using a single type of EHR data (ie, diagnosis codes) [27-29]. To avoid this bias, the Electronic Medical Records and Genomics (eMERGE) consortium [30,31] has piloted the development of EHR-based phenotyping algorithms using multiple types of EHR data [32-34]. This has given rise to a number of phenotyping algorithms that use both structured EHR data (eg, demographics, diagnosis and procedure codes, laboratory test results, and medications) and unstructured EHR data (eg, clinical notes, imaging reports, and discharge summaries) [35-38]. However, the eMERGE consortium algorithms are typically focused on identifying cases and noncases rather than characterizing a new-onset (ie, incident) disease in a population. Moreover, extracting information from unstructured clinical text is a nontrivial task that involves natural language processing techniques [39-41].

In our paper, we address existing challenges for stroke ascertainment, specifically for incident stroke. Our research objective is to develop and validate a machine learning–based phenotyping algorithm to identify incident stroke and detailed stroke subtypes based on three major EHR-derived data elements: clinical concepts extracted from clinical notes; ICD, Ninth Revision (ICD-9) diagnosis codes; and CPT procedure codes.

## Methods

This study was approved by the Mayo Clinic Institutional Review Board (no. 17-008818) and is in accordance with the ethical standards mandated by the committee on responsible human experimentation. The data that support the findings of this study are available from the corresponding author upon reasonable request.

### Study Design

This was a predictive modeling study that used observational cohort data for training and validation. We employed an atrial fibrillation (AF) cohort, in which all incidences of stroke were manually ascertained in a previous study [4], to train and test our phenotyping algorithm for the date of incident stroke events. We then evaluated the generalizability of our algorithm in a general population cohort.

### The AF Cohort

The AF cohort comprised a patient population from Olmsted County, Minnesota, USA [4,42]. Olmsted County is an area relatively isolated from other urban centers with only a few providers delivering most care to residents, primarily Mayo Clinic and Olmsted Medical Center [43-45]. Extracting all health care–related events was completed through the Rochester Epidemiology Project (REP), a records linkage system [43,44]. The REP is a records linkage system that allows retrieval of nearly all health care utilization and outcomes of residents living in Olmsted County. The electronic indexes of the REP include demographic information, diagnostic and procedure codes, health care utilization data, outpatient drug prescriptions, results of laboratory tests, and information about smoking, height, weight, and body mass index. ICD-9 codes and the Mayo Clinic electrocardiograms were obtained among adults aged ≥18 years from 2000 to 2014 to ascertain AF. Patients were identified by the presence of an ICD-9 code for stroke through March 31, 2015, and then validated by manual review of the EHR. Strokes were classified as ischemic strokes/transient ischemic attack or hemorrhagic strokes [4,46]. The first (incident) event of each type of stroke after the incident AF date was ascertained, regardless of whether a patient had a prior stroke. The AF cohort included 4914 validated patients with AF, 1773 of whom were screened for a possible stroke. Table 1 shows the cohort characteristics. Manual abstraction of the EHR validated the stroke code in 740 patients. Manual ascertainment of stroke and the dates of the events were used as a gold standard to train and test the stroke algorithm.

**Table 1:** Atrial fibrillation cohort characteristics.

Measure		Cohort (n=4914)	Screened (n=1773)
**Gender, n (%)**			
	Female	2309 (46.99)	869 (49.01)
	Male	2605 (53.01)	904 (50.99)
**Age at diagnosis of AF^a^ (years), mean**			
	Female	76	80
	Male	70	74
ICD-9^b^ diagnosis codes^c^, n		27,243	27,243

^a^AF: atrial fibrillation.

^b^ICD-9: International Classification of Diseases, Ninth Revision.

^c^ICD retrieval was from AF incidence date to March 31, 2015. AF validations were from 2000 to 2014.

### Candidate Predictive Features

The proposed algorithm aimed to identify first (incident) stroke events within a certain time frame. The three major data elements we used were clinical concepts, ICD-9 codes, and CPT codes. To align with the manual review process, only codes and clinical notes from the AF incident date to March 31, 2015, were retrieved and processed. In our analyses, we constructed different models by varying the inclusion of CPT codes and symptom-related clinical concepts in the model feature set and compared different models’ performances.

Both ICD-9 and CPT codes were extracted from the REP database. Clinical concepts were identified from the major and secondary problem list section of Mayo Clinic EHR, and from clinical notes from other REP sites using a natural language processing system, MedTagger [47]. Expert-provided vocabulary was adopted from a previous study [48] to extract clinical concepts from unstructured clinical notes. MedTagger enables a series of natural language processing processes, including regular expression matching and positive, negative, or probable identification with ConText [49,50], and is insensitive to upper and lower case. MedTagger is also able to determine if the extracted clinical concepts are referring to the patients or their family members, or if the extracted clinical concepts are in present tense and thus are referring to a current event rather than a past medical condition. We considered only documents with positive, present-tense stroke mentions that were referring to patients themselves. Table S1 in Multimedia Appendix 1 lists clinical concepts for 2 major stroke subtypes and stroke-related symptoms. Table S2 in Multimedia Appendix 1 lists ICD-9 codes for 2 stroke subtypes and stroke-related symptoms. Table S3 in Multimedia Appendix 1 lists the CPT codes used in the stroke algorithm.

**Figure 1:**
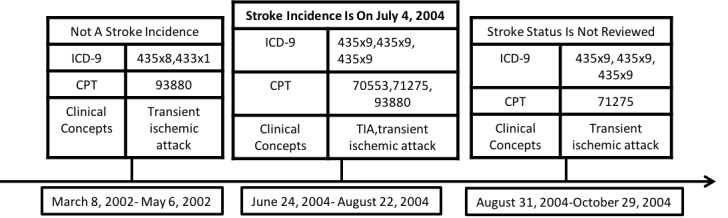
Inclusion of clinical concepts and codes on a patient visit timeline. CPT: Current Procedural Terminology; ICD-9: International Classification of Diseases, Ninth Revision.

Clinical concept dates were determined by the date of the clinical notes from which clinical concepts were extracted, while ICD-9 and CPT code dates were extracted from the REP. Each visit was characterized by clinical concepts, ICD-9, and CPT codes within a 60-day window. The visit date was determined by the earliest date of any of the 3 elements in the 60-day window. If visit dates were within a 60-day window of a confirmed stroke incidence date, they were considered positive instances; otherwise, they were considered negative instances. Figure 1 demonstrates an example with an incident stroke on July 4, 2004. All visits were extracted and included in our data set if there was at least one key word or code during a 60-day window. Nurse abstractors reviewed every visit sequentially until they determined the incidence date to be July 4, 2004. All subsequent visits after a positive stroke incident were not reviewed and thus were not included in our analyses. Since the confirmed stroke incidence date fell in the date range of the third visit (June 24, 2004-August 22, 2004), we considered the combination of codes and clinical concepts in this visit to be predictive of a positive stroke incidence.

### Data Analysis

**Figure 2:**
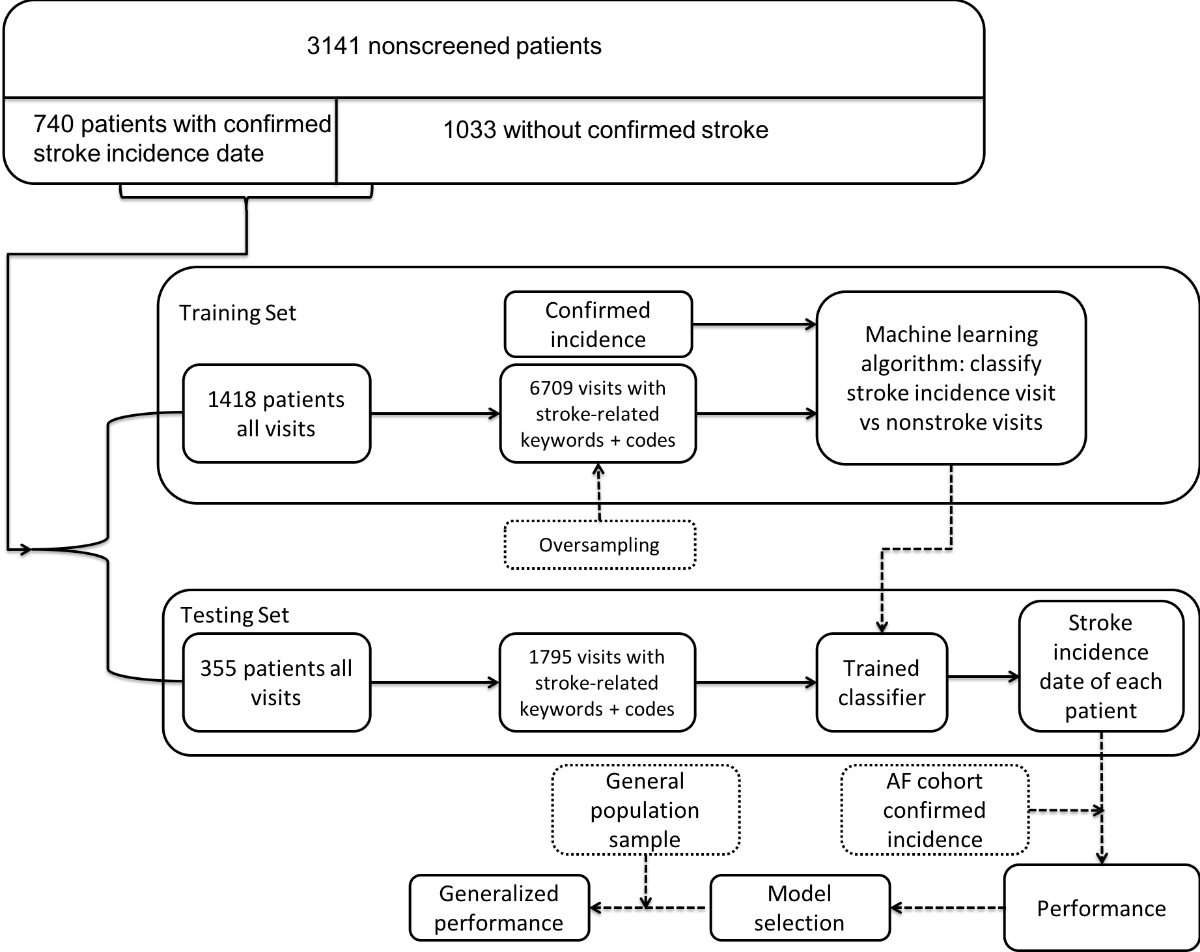
Stroke algorithm training and testing workflow. AF: atrial fibrillation.

After incident stroke was confirmed, visits afterwards were not reviewed by abstractors and thus excluded from our overall data set. Figure 2 shows the workflow of the algorithm training and testing process. We created a data set with 9130 confirmed visits (with stroke vs nonstroke labels) among the 1773 patients. In total, there were 746 stroke visits and 8384 nonstroke visits. The stroke incidence count (n=746) was larger than the number of patients with confirmed stroke incidence (740) because incidence dates for different subtypes of stroke (ischemic stroke/ transient ischemic attack and hemorrhagic stroke) were all recorded, such that patients might have had multiple incidence dates. We included data from a randomly selected 79.98% of our screened patients (1418/1773 patients; 7253 visits) as a training set and the remaining 20.02% of our screened patients (355/1773 patients; 1877 visits) were retained as an independent testing set. Due to the outcome imbalance in the data set (positive:negative ratio of about 1:10), we used the synthetic minority oversampling technique [51] to create oversampled training data sets with an oversampling percentage of 1000%.

We considered two machine learning classifiers, logistic regression and random forest [52], to train our phenotyping models. Logistic regression served as a baseline modeling algorithm. Random forest was also chosen because of its high performance with structured input features and better model flexibility. We also considered the influence of feature groups by varying the inclusion of CPT codes and symptom terms in the input feature set. The hyperparameter tuning of the machine learning models was performed using 10-fold cross-validation. The performance metrics adopted for the machine learning task in the test set were precision, recall, and F score. The oversampling and machine learning modeling training and testing processes were implemented in Weka 3 (University of Waikato) [53]. Additional statistical summaries were performed using the R statistical software version 3.6.2 (The R Foundation for Statistical Computing). Quantitative variables are summarized as means, while nominal variables are expressed by counts and percentages.

### Validation Cohort

We evaluated the generalizability of our model on a sample from a general population cohort of 71,429 patients. This cohort consisted of individuals sampled in Olmsted County, Minnesota on January 1, 2006, with an age ≥30 years and with no prior history of cardiovascular disease. We applied the best performing model based on the leave-out test set to this entire population cohort to generate incident stroke predictions. We then randomly selected 50 patients from those who had no stroke-related features (ie, de facto negative stroke predictions), 50 patients from those who were shown to have negative stroke predictions, and 50 patients from those who were shown to have positive stroke predictions and a predicted incident stroke for evaluation. This verification-based sampling strategy allowed for estimates of positive and negative predictive values (PPVs and NPVs, respectively) by conditioning on algorithm predictions. Under these conditions (n=50), the half-width of the 95% Wilson score CI for the PPVs and separate NPVs would be approximately 0.1 for a true value of 0.85.

All 150 patient cases were reviewed by 1 nurse abstractor to confirm incident stroke, which served as our gold standard. We recorded model prediction outputs on all patient visits in the 150-patient validation set. We combined visit-level true predictions to generate patient-level incidence predictions by saving only the earliest date of positive predictions as stroke incidences. We compared patient-level incidence predictions with our gold standard. True prediction in our evaluation meant the date of the predicted incident stroke was within 60 days of the abstracted stroke date. A 2 x 2 confusion matrix was used to calculate performance scores for prediction evaluation. Model performance metrics included PPV and NPV using manual evaluation as the gold standard and patient-level predictions to calculate true positives, false positives, true negatives, and false negatives. The uncertainty of these performance estimates was calculated using Wilson score 95% CI for proportions.

In addition, we developed heuristic rules to distinguish stroke subtype (ischemic stroke/transient ischemic attack or hemorrhagic stroke) of each identified stroke incidence by analyzing the composition of keyword or code input feature sets (in a window of 60 days). We counted the number of keywords or codes for each ischemic stroke/transient ischemic attack and hemorrhagic stroke. If an input feature set contained more keywords or codes for ischemic stroke/transient ischemic attack, then this incidence was considered an ischemic stroke incidence; otherwise, it was considered a hemorrhagic stroke incidence. We only evaluated correct incident stroke predictions from the previous step in the evaluation data set with manually ascertained subtypes as the gold standard. Accuracy was calculated to measure performance of the subtype identification.

## Results

### Model Selection and Subtype Identification

Table 2 shows the algorithm performance measured on the test set for 8 models run on 4 input combinations and 2 classifiers (logistic regression and random forest). The random forest classifier outperformed the logistic classifier regardless of the feature sets used. Inclusion of CPT codes as features improved the performance for the random forest model with F score increased from 0.836 (Model 3) to 0.905 (Model 1). However, in the logistic model, the inclusion of CPT codes slightly improved the F score from 0.772 (Model 4) to 0.793 (Model 2). Using comparisons to all features (Model 1 and 2) and excluding the symptom terms (Model 6 and 7) achieved better F score (values italicized in Table 2).

**Table 2:** Stroke algorithm performance.

Model	ICD-9^a^	Clinical concept	CPT^b^	Classifier	Precision	Recall	F score
1	Yes	Symptoms + disease concepts	Yes	Random forest	0.912	0.906	0.905
2	Yes	Symptoms + disease concepts	Yes	Logistic	0.807	0.795	0.793
3	Yes	Symptoms + disease concepts	No	Random forest	0.835	0.845	0.836
4	Yes	Symptoms + disease concepts	No	Logistic	0.791	0.777	0.772
5	Yes	Disease-only concept	Yes	Random forest	*0.920*	*0.915*	*0.915*
6	Yes	Disease-only concept	Yes	Logistic	0.809	0.798	0.796
7	Yes	Disease-only concept	No	Random forest	0.856	0.847	0.846
8	Yes	Disease-only concept	No	Logistic	0.779	0.767	0.763

^a^ICD-9: International Classification of Diseases, Ninth Revision.

^b^CPT: Current Procedural Terminology.

### Model Generalizability

Table 3 shows the distribution of stroke features in the AF cohort and the general population cohort. The AF cohort had a higher proportion of stroke-related codes and concepts. Results from the evaluation of the 150 selected patient records are presented in Table 4. Prediction performance corresponded to a PPV of 0.86 (95% CI 0.74-0.93), an NPV without ICD codes of 1.00 (95% CI 0.92-1.00), and an NPV with codes of 0.92 (95% CI 0.90-0.98). No strokes were observed among patients with no eligible stroke ICD codes. For subtype characterization, we achieved an accuracy of 80% (95% CI 0.68-0.89) in the general population sample.

**Table 3:** Patient feature distribution post-AF.

Stroke feature distribution	AF^a^ screened		AF nonscreened (n=3141), n (%)		AF cohort total (n=4914), n (%)		Olmsted County cohort (N=71,429), n (%)	
	Stroke (n=740), n (%)	No stroke (n=1033), n (%)						
ICD-9^b^+CPT^c^+CC^d^	654 (88.37)	379 (36.69)	0 (0)		1033 (21.02)		2726 (3.82)	
ICD-9+CPT	66 (8.92)	596 (57.70)	0 (0)		662 (13.47)		1018 (1.42)	
ICD-9+CC	9 (1.22)	12 (1.16)	0 (0)		21 (0.43)		48 (0.067)	
CPT+CC	0 (0)	0 (0)	167 (5.32)		167 (3.40)		1595 (2.23)	
ICD-9	11 (1.49)	46 (4.45)	0 (0)		57 (1.16)		194 (0.27)	
CPT	0 (0)	0 (0)	1736 (55.27)		1736 (35.33)		17,433 (24.40)	
CC	0 (0)	0 (0)	11 (0.35)		11 (0.24)		566 (0.79)	
None	0 (0)	0 (0)	1227 (39.06)		1227 (24.97)		47,849 (66.99)	

^a^AF: atrial fibrillation.

^b^ICD-9: International Classification of Diseases, Ninth Revision.

^c^CPT: Current Procedural Terminology.

^d^CC: clinical concepts.

**Table 4:** Generalizability analysis results from the Olmsted County cohort.

Gold standard	Stroke algorithm prediction (N=150)		
	Negative (n=100)		Positive (n=50)
	No ICD-9^a^ codes (n=50)	Predicted no stroke (n=50)	
Stroke	0	4	43
No Stroke	50	46	7

^a^ICD-9: International Classification of Diseases, Ninth Revision.

## Discussion

### Principal Findings

The rapid expansion of information available in EHRs opens new opportunities to combine structured and unstructured data for research. Advances in machine learning methods and tools facilitate the combination of multimodal clinical data for effective development of phenotyping algorithms. However, performance of stroke electronic phenotyping algorithms varies by stroke subtypes [25] and phenotyping tasks (ie, case vs noncase or incident stroke phenotyping). Our previous study showed that when naïve ICD codes with clinical concept matching were used, stroke incidence identification had a PPV of 60.6% while case-versus-noncase identification had a much higher PPV of 88.7% [20].

In this study, we included clinical concepts extracted from clinical notes along with ICD-9 and CPT codes for incident stroke ascertainment. The rationale to add CPT codes is that diagnosis of stroke usually needs to be confirmed by imaging evidence and will probably be followed by therapeutic procedures. Thus, the addition of CPT codes in the model could potentially help to reduce the information redundancy effect by distinguishing between past and current events recorded in clinical notes. Our algorithm closely resembles the ascertainment process (chart review) of clinicians, which uses multiple types of EHR data (eg, diagnoses and procedure codes, unstructured clinical notes) in a parsimonious manner. Due to the redundancy and temporal ambiguity in unstructured clinical notes, we needed to construct a data set with sufficient and interpretable features from multimodal clinical data.

We found that the random forest generated better results, while the addition of CPT codes improved overall performance. This may be because imaging procedures, especially head CT or MRI, are critical in the diagnosis of stroke. Therefore, CPT codes of such procedures can be important indicators for distinguishing between incident and historical events. In addition, ICD codes and therapeutic procedures can vary significantly between incident and recurrent events. Meanwhile, we observed that the additions of stroke-related symptom concepts were not helpful for the phenotyping task. This may be due to the fact that our stroke incidence ascertainment depends largely on the ubiquitous nature of many stroke-related symptoms: they may be stroke-related but not necessarily stroke specific. Additionally, ascertainment requires well-documented evidence, such as imaging or imaging reports. Without properly recorded evidence, patients are not likely to be ascertained as stroke.

Our generalizability evaluation demonstrates that models trained using a specific disease cohort for incident stroke ascertainment can generalize well to a general patient population. This is very encouraging given there are many existing patient cohorts available. Secondary use of these patient cohorts would be a cost-effective way for developing machine learning–based phenotyping algorithms. The study also illustrates that incorporating structured EHR data, such as CPT codes, can effectively distinguish incident stroke mentions from historical events in the clinical notes.

One limitation of our study is the dependence of domain experts to provide relevant clinical concepts, ICD-9 codes, and CPT codes. In the future, we will explore advance feature engineering approaches to identify those relevant concepts or codes automatically or semiautomatically. We are also aware that our imbalance cohort data and oversampling strategies might have introduced overfitting. Although our evaluation in the general population proved the performance of the algorithm, in the future, we can adopt a case–control matching strategy to deal with imbalanced data and mitigate the potential overfitting issue. In addition, new treatment strategies (mechanical thrombectomy) to treat stroke have been in the market in recent years, and thus the features used in our algorithm could have different weights for predictions of events in different temporal settings. A more precise strategy could consider using different features for prediction tasks in different time frames, where variations in clinical knowledge and care path have been considered.

### Conclusions

In conclusion, the high prevalence of stroke and the lack of an efficient algorithm to confirm incident stroke events necessitate the development of an effective and interpretable algorithm to identify incident stroke occurrences. In this paper, we described our efforts to develop and validate an EHR-based algorithm that accurately identifies incident stroke events and goes beyond typical case-versus-noncase stroke identification. Our algorithm’s good performance in a general population sample demonstrates its generalizability and potential to be adopted by other institutions.

## Appendix

Multimedia Appendix 1 ICD-9, CPT codes, and clinical concepts for two major stroke subtypes and stroke-related symptoms.

## Abbreviations

AF: atrial fibrillation
CPT: Current Procedural Terminology
CT: computed tomography
EHR: electronic health record
eMERGE: Electronic Medical Records and Genomics
ICD: International Classification of Diseases
ICD-9: International Classification of Diseases, Ninth Revision
MRI: magnetic resonance imaging
NPV: negative predictive value
PPV: positive predictive value
REP: Rochester Epidemiology Project

## References

[1] Bonita R (1992). Epidemiology of stroke. Lancet.

[2] Clinical diagnosis of stroke subtypes. UptoDate.

[3] Morley KI, Wallace J, Denaxas SC, Hunter RJ, Patel RS, Perel P, Shah AD, Timmis AD, Schilling RJ, Hemingway H (2014). Defining disease phenotypes using national linked electronic health records: a case study of atrial fibrillation. PLoS One.

[4] Chamberlain AM, Brown RD, Alonso A, Gersh BJ, Killian JM, Weston SA, Roger VL (2016). No decline in the risk of stroke following incident atrial fibrillation since 2000 in the community: a concerning trend. J Am Heart Assoc.

[5] Scannapieco FA, Bush RB, Paju S (2003). Associations between periodontal disease and risk for atherosclerosis, cardiovascular disease, and stroke. A systematic review. Ann Periodontol.

[6] Slee VN (1978). The International Classification of Diseases: ninth revision (ICD-9). Ann Intern Med.

[7] Imran Tasnim F, Posner Daniel, Honerlaw Jacqueline, Vassy Jason L, Song Rebecca J, Ho Yuk-Lam, Kittner Steven J, Liao Katherine P, Cai Tianxi, O'Donnell Christopher J, Djousse Luc, Gagnon David R, Gaziano J Michael, Wilson Peter Wf, Cho Kelly (2018). A phenotyping algorithm to identify acute ischemic stroke accurately from a national biobank: the Million Veteran Program. Clin Epidemiol.

[8] Coull A, Silver L, Bull L, Giles M, Rothwell P, Oxford Vascular (OXVASC) Study (2004). Direct assessment of completeness of ascertainment in a stroke incidence study. Stroke.

[9] Thangaraj P, Kummer B, Lorberbaum T, Elkind M, Tatonetti N (2020). Comparative analysis, applications, and interpretation of electronic health record-based stroke phenotyping methods. BioData Min.

[10] Moons KGM, Bots ML, Salonen JT, Elwood PC, Freire de Concalves A, Nikitin Y, Sivenius J, Inzitari D, Benetou V, Tuomilehto J, Koudstaal PJ, Grobbee DE (2002). Prediction of stroke in the general population in Europe (EUROSTROKE): Is there a role for fibrinogen and electrocardiography?. J Epidemiol Community Health.

[11] Ho King Chung, Speier William, El-Saden Suzie, Liebeskind David S, Saver Jeffery L, Bui Alex A T, Arnold Corey W (2014). Predicting discharge mortality after acute ischemic stroke using balanced data. AMIA Annu Symp Proc.

[12] Peng S, Chuang Y, Kang T, Tseng K (2010). Random forest can predict 30-day mortality of spontaneous intracerebral hemorrhage with remarkable discrimination. Eur J Neurol.

[13] Colak C, Karaman E, Turtay MG (2015). Application of knowledge discovery process on the prediction of stroke. Comput Methods Programs Biomed.

[14] Asadi H, Dowling R, Yan B, Mitchell P (2014). Machine learning for outcome prediction of acute ischemic stroke post intra-arterial therapy. PLoS One.

[15] Denny JC, Crawford DC, Ritchie MD, Bielinski SJ, Basford MA, Bradford Y, Chai HS, Bastarache L, Zuvich R, Peissig P, Carrell D, Ramirez AH, Pathak J, Wilke RA, Rasmussen L, Wang X, Pacheco JA, Kho AN, Hayes MG, Weston N, Matsumoto M, Kopp PA, Newton KM, Jarvik GP, Li R, Manolio TA, Kullo IJ, Chute CG, Chisholm RL, Larson EB, McCarty CA, Masys DR, Roden DM, de Andrade M (2011). Variants near FOXE1 are associated with hypothyroidism and other thyroid conditions: using electronic medical records for genome- and phenome-wide studies. Am J Hum Genet.

[16] Conway M, Berg R, Carrell D, Denny J, Kho A, Kullo I, Linneman James G, Pacheco Jennifer A, Peissig Peggy, Rasmussen Luke, Weston Noah, Chute Christopher G, Pathak Jyotishman (2011). Analyzing the heterogeneity and complexity of Electronic Health Record oriented phenotyping algorithms. AMIA Annu Symp Proc.

[17] Overby CL, Pathak J, Gottesman O, Haerian K, Perotte A, Murphy S, Bruce K, Johnson S, Talwalkar J, Shen Y, Ellis S, Kullo I, Chute C, Friedman C, Bottinger E, Hripcsak G, Weng C (2013). A collaborative approach to developing an electronic health record phenotyping algorithm for drug-induced liver injury. J Am Med Inform Assoc.

[18] Castro VM, Minnier J, Murphy SN, Kohane I, Churchill SE, Gainer V, Cai T, Hoffnagle AG, Dai Y, Block S, Weill SR, Nadal-Vicens M, Pollastri AR, Rosenquist JN, Goryachev S, Ongur D, Sklar P, Perlis RH, Smoller JW, International Cohort Collection for Bipolar Disorder Consortium (2015). Validation of electronic health record phenotyping of bipolar disorder cases and controls. Am J Psychiatry.

[19] Feigin Valery L, Carter Kristie (2004). Editorial comment--Stroke incidence studies one step closer to the elusive gold standard?. Stroke.

[20] Hollands GJ, Marteau TM, Fletcher PC (2016). Non-conscious processes in changing health-related behaviour: a conceptual analysis and framework. Health Psychology Review.

[21] Ni Y, Alwell K, Moomaw CJ, Woo D, Adeoye O, Flaherty ML, Ferioli S, Mackey J, De Los Rios La Rosa F, Martini S, Khatri P, Kleindorfer D, Kissela BM (2018). Towards phenotyping stroke: leveraging data from a large-scale epidemiological study to detect stroke diagnosis. PLoS One.

[22] Howard VJ, McClure LA, Meschia JF, Pulley L, Orr SC, Friday GH (2006). High prevalence of stroke symptoms among persons without a diagnosis of stroke or transient ischemic attack in a general population: the REasons for Geographic And Racial Differences in Stroke (REGARDS) study. Arch Intern Med.

[23] How a CPT code becomes a code. American Speech-Language-Hearing Association.

[24] Denaxas SC, George J, Herrett E, Shah AD, Kalra D, Hingorani AD, Kivimaki M, Timmis AD, Smeeth L, Hemingway H (2012). Data resource profile: cardiovascular disease research using linked bespoke studies and electronic health records (CALIBER). Int J Epidemiol.

[25] Woodfield R, Grant I, Sudlow CLM, UK Biobank Stroke Outcomes Group, UK Biobank Follow-Up and Outcomes Working Group (2015). Accuracy of electronic health record data for identifying stroke cases in large-scale epidemiological studies: a systematic review from the UK Biobank Stroke Outcomes Group. PLoS One.

[26] Schuemie MJ, Sen E, 't JGW, van SEM, Sturkenboom MC, Kors JA (2012). Automating classification of free-text electronic health records for epidemiological studies. Pharmacoepidemiol Drug Saf.

[27] Schellenbaum GD, Heckbert SR, Smith NL, Rea TD, Lumley T, Kitzman DW, Roger VL, Taylor HA, Psaty BM (2006). Congestive heart failure incidence and prognosis: case identification using central adjudication versus hospital discharge diagnoses. Ann Epidemiol.

[28] Pakhomov Serguei, Weston Susan A, Jacobsen Steven J, Chute Christopher G, Meverden Ryan, Roger Véronique L (2007). Electronic medical records for clinical research: application to the identification of heart failure. Am J Manag Care.

[29] Ermenc B (1999). Minimizing mistakes in clinical diagnosis. J Forensic Sci.

[30] McCarty CA, Chisholm RL, Chute CG, Kullo IJ, Jarvik GP, Larson EB, Li R, Masys DR, Ritchie MD, Roden DM, Struewing JP, Wolf WA, eMERGE Team (2011). The eMERGE Network: a consortium of biorepositories linked to electronic medical records data for conducting genomic studies. BMC Med Genomics.

[31] Gottesman O, Kuivaniemi H, Tromp G, Faucett WA, Li R, Manolio TA, Sanderson SC, Kannry J, Zinberg R, Basford MA, Brilliant M, Carey DJ, Chisholm RL, Chute CG, Connolly JJ, Crosslin D, Denny JC, Gallego CJ, Haines JL, Hakonarson H, Harley J, Jarvik GP, Kohane I, Kullo IJ, Larson EB, McCarty C, Ritchie MD, Roden DM, Smith ME, Böttinger EP, Williams MS, e M (2013). The Electronic Medical Records and Genomics (eMERGE) Network: past, present, and future. Genet Med.

[32] Denny JC (2012). Chapter 13: Mining electronic health records in the genomics era. PLoS Comput Biol.

[33] Kho AN, Hayes MG, Rasmussen-Torvik L, Pacheco JA, Thompson WK, Armstrong LL, Denny JC, Peissig PL, Miller AW, Wei W, Bielinski SJ, Chute CG, Leibson CL, Jarvik GP, Crosslin DR, Carlson CS, Newton KM, Wolf WA, Chisholm RL, Lowe WL (2012). Use of diverse electronic medical record systems to identify genetic risk for type 2 diabetes within a genome-wide association study. J Am Med Inform Assoc.

[34] Ritchie MD, Denny JC, Zuvich RL, Crawford DC, Schildcrout JS, Bastarache L, Ramirez AH, Mosley JD, Pulley JM, Basford MA, Bradford Y, Rasmussen LV, Pathak J, Chute CG, Kullo IJ, McCarty CA, Chisholm RL, Kho AN, Carlson CS, Larson EB, Jarvik GP, Sotoodehnia N, Manolio TA, Li R, Masys DR, Haines JL, Roden DM, Cohorts for HeartAging Research in Genomic Epidemiology (CHARGE) QRS Group (2013). Genome- and phenome-wide analyses of cardiac conduction identifies markers of arrhythmia risk. Circulation.

[35] Wright A, Pang J, Feblowitz JC, Maloney FL, Wilcox AR, Ramelson HZ, Schneider LI, Bates DW (2011). A method and knowledge base for automated inference of patient problems from structured data in an electronic medical record. J Am Med Inform Assoc.

[36] Garvin JH, DuVall SL, South BR, Bray BE, Bolton D, Heavirland J, Pickard S, Heidenreich P, Shen S, Weir C, Samore M, Goldstein MK (2012). Automated extraction of ejection fraction for quality measurement using regular expressions in Unstructured Information Management Architecture (UIMA) for heart failure. J Am Med Inform Assoc.

[37] Percha B, Nassif H, Lipson J, Burnside E, Rubin D (2012). Automatic classification of mammography reports by BI-RADS breast tissue composition class. J Am Med Inform Assoc.

[38] Jiang M, Chen Y, Liu M, Rosenbloom ST, Mani S, Denny JC, Xu H (2011). A study of machine-learning-based approaches to extract clinical entities and their assertions from discharge summaries. J Am Med Inform Assoc.

[39] Xu Y, Hong K, Tsujii J, Chang EI (2012). Feature engineering combined with machine learning and rule-based methods for structured information extraction from narrative clinical discharge summaries. J Am Med Inform Assoc.

[40] D'Avolio LW, Nguyen TM, Goryachev S, Fiore LD (2011). Automated concept-level information extraction to reduce the need for custom software and rules development. J Am Med Inform Assoc.

[41] Kahn MG, Weng C (2012). Clinical research informatics: a conceptual perspective. J Am Med Inform Assoc.

[42] Chamberlain AM, Gersh BJ, Alonso A, Chen LY, Berardi C, Manemann SM, Killian JM, Weston SA, Roger VL (2015). Decade-long trends in atrial fibrillation incidence and survival: a community study. Am J Med.

[43] St Sauver JL, Grossardt BR, Yawn BP, Melton LJ, Rocca WA (2011). Use of a medical records linkage system to enumerate a dynamic population over time: the Rochester epidemiology project. Am J Epidemiol.

[44] Rocca WA, Yawn BP, St Sauver JL, Grossardt BR, Melton LJ (2012). History of the Rochester Epidemiology Project: half a century of medical records linkage in a US population. Mayo Clin Proc.

[45] Rocca WA, Grossardt BR, Brue SM, Bock-Goodner CM, Chamberlain AM, Wilson PM, Finney Rutten LJ, St Sauver JL (2018). Data Resource Profile: Expansion of the Rochester Epidemiology Project medical records-linkage system (E-REP). Int J Epidemiol.

[46] Witt BJ, Brown RD, Jacobsen SJ, Weston SA, Yawn BP, Roger VL (2005). A community-based study of stroke incidence after myocardial infarction. Ann Intern Med.

[47] Liu H, Bielinski SJ, Sohn S, Murphy S, Wagholikar KB, Jonnalagadda SR, Ravikumar K E, Wu Stephen T, Kullo Iftikhar J, Chute Christopher G (2013). An information extraction framework for cohort identification using electronic health records. AMIA Jt Summits Transl Sci Proc.

[48] Mitchell-Box K, Braun KL (2012). Fathers' thoughts on breastfeeding and implications for a theory-based intervention. J Obstet Gynecol Neonatal Nurs.

[49] Harkema H, Dowling JN, Thornblade T, Chapman WW (2009). ConText: an algorithm for determining negation, experiencer, and temporal status from clinical reports. J Biomed Inform.

[50] Chapman W, Chu D, Dowling J (2007). editors. ConText: An algorithm for identifying contextual features from clinical text.

[51] Chawla Nv, Bowyer Kw, Hall Lo, Kegelmeyer Wp (2002). SMOTE: Synthetic Minority Over-sampling Technique. JAIR.

[52] Liaw A, Weiner M (2002). Classification and regression by randomForest. R news.

[53] Eibe F, Hall M, Witten I (2016). The WEKA Workbench. Online Appendix for Data Mining: Practical Machine Learning Tools and Techniques. Morgan Kaufmann.

