# Supplementary material for: Natural Language Processing and Machine Learning for Identifying Incident Stroke From Electronic Health Records: Algorithm Development and Validation
Source: J Med Internet Res. 2021 Mar 8;23(3):e22951. doi: 10.2196/22951 (PMC7985804; doi:10.2196/22951)
Supplement: Multimedia Appendix 1 [file jmir_v23i3e22951_app1.docx]

**MULTIMEDIA APPENDIX**

Using Natural Language Processing and Machine Learning to Identify Incident Stroke from Electronic Health Records

Yiqing Zhao,^1^ PhD; Sunyang Fu, ^1^ MHI; Suzette J. Bielinski, ^1^ PhD, MEd; Paul A. Decker, ^1^ MS; Alanna M. Chamberlain, ^1^ PhD; Veronique L. Roger,^1^ MD; Hongfang Liu,^1^ PhD; Nicholas B. Larson,^1^ PhD

^1^Department of Health Sciences Research, Mayo Clinic, 200 First Street SW, Rochester, MN, United States

^2^Department of Cardiovascular Diseases (V.L.R.), Mayo Clinic, 200 First Street SW, Rochester, MN, United States

| **Table 1 in Appendix.** Expert provided keywords for stroke related symptoms and two stroke subtypes. | | |
| --- | --- | --- |
| Ischemic Stroke/Transient Ischemic Attacks (TIA) | Hemorrhage | Symptoms |
| stroke  acute stroke  chronic stroke  ischemic stroke  acute ischemic stroke  ais  cerebral (infarct\|infarction)  acute cerebral (infarct\|infarction)  brain (infarct\|infarction)  acute brain (infarct\|infarction)  cerebral embolism  cerebral thrombosis  retinal artery occlusion  retinal artery embolism  retinal artery thrombosis  central retinal artery occlusion  central retinal artery embolism  central retinal artery thrombosis  branch retinal artery occlusion  branch retinal artery embolism  branch retinal artery thrombosis  crao  brao  spinal cord stroke  spinal cord (infarct\|infarction)  transient ischemic attack  tia  cerebrovascular accident | epidural hemorrhage  edh  subdural hemorrhage  sdh  subarachnoid hemorrhage  sah  intracerebral hemorrhage  ich  intraparenchymal hemorrhage  iph  intraventricular hemorrhage  ivh | aphasia  hemiplegia  hemiparesis  arm weakness  leg weakness  hemisensory loss  face numbness  arm numbness  leg numbness  hemiataxia  arm ataxia  leg ataxia  gait ataxia  hemineglect  visual field defect  quadrantanopia  hemianopia  amaurosis fugax  monocular vision loss  diplopia  dysarthria  dysphagia  apraxia  vertigo |

| **Table 2 in Appendix.** ICD-9 codes for two stroke subtypes. | | |
| --- | --- | --- |
| Ischemic Stroke/Transient Ischemic Attacks (TIA) | | Hemorrhage |
| 433  433.0  433.01  433.1  433.10  433.11  433.2  433.21  433.3  433.31  433.8  433.81  433.9  433.91 | 434  434.0  434.01  434.1  434.11  434.9  434.91  435  435.0  435.1  435.2  435.3  435.8  435.9  436 | 430  431  432  432.0  432.1  432.9 |

| **Table 3 in Appendix.** CPT codes for stroke algorithm. | |
| --- | --- |
|  | CPT |
| Head CT | 70450 (noncontrast), 70460 (with contrast), 70470 (with and without contrast) |
| Brain MRI | 70551 (noncontrast), 70552 (with contrast), 70553 (with and without contrast) |
| Head MR angiography | 70544, 70545 |
| CT angiography | 70496, 70498, 71275, 72191, 73206, 73706, 74174, 74175, 75635 |
| Cerebral angiography | 36221, 36222, 36223, 36224, 36225, 36226, 36227, 36228 |
| Carotid ultrasound | 93880, 93882 |
| Intubation (ED) | 31500 |
| Intraventricular drain | 62160 |
| Thrombolytic therapy | 37187, 37188, 37195, 37211, 37212, 37213, 37214, 61645 |
| Aneurysm clipping | 61697, 61698, 61700, 61702 |
| Aneurysm coiling | 61624, 75894 |
| AVM resection | 61680, 61682, 61684, 61686, 61690, 61692, 61697, 61698, 61700, 61702, 61703, 61705, 61708, 61710 |
| Hemicraniectomy | 61322, 61323 |
| Carotid endarterectomy | 35301 |
| Carotid stent placement | 37215, 37216, 37217, 37218, 61635 |
| Balloon angioplasty | 61630 |
